# Fracture Risk of Endodontic Files: Clinical Analysis of Reciproc and X1 Blue After Multiple Uses

**DOI:** 10.1111/aej.70003

**Published:** 2025-08-06

**Authors:** Elisa Korte Fortes Gollo, Fábio de Almeida Gomes, Katerine Jahnecke Pilownic, Daiana Elisabeth Böttcher, Carolina Clasen Vieira, Fernanda Geraldo Pappen

**Affiliations:** ^1^ Post‐Graduate Program in Dentistry Federal University of Pelotas, UFPel Pelotas Rio Grande do Sul Brazil; ^2^ School of Dentistry Universidade de Fortaleza, UNIFOR Fortaleza Ceará Brazil; ^3^ Post‐Graduate Program in Dentistry, School of Health and Life Science Pontifical Catholic University of Rio Grande Do Sul, PUCRS Porto Alegre Rio Grande do Sul Brazil

**Keywords:** endodontics, file fracture, observational study, Reciproc, X1 Blue

## Abstract

This retrospective study assessed the fracture incidence of Reciproc R25 and X1 Blue 25.06 files after up to three uses in 1720 root canals (1620 teeth) treated by a specialist. A standardised protocol was followed for all procedures. Periapical radiographs with the fractured instrument were used to evaluate the fragment's location and size and to determine the root curvature's angle. Data included fracture site (apical, middle, coronal) and management (removal, bypass or retention). Files were inspected post‐use; non‐deformed ones were sterilised and reused. Descriptive statistical analysis was performed using STATA 14. Among 1317 canals treated with R25 and 403 with X1 Blue, six fractures occurred: four R25 (0.3%) and two X1 Blue (0.5%). Four fractures were in molars, all in the apical third. Two fragments were bypassed, one removed, and three retained. Results indicate low fracture rates for both file systems, even with repeated use in primary treatments.

## 1 Introduction

Pursuing excellence in root canal treatment has led to a paradigm shift from manual instrumentation with stainless steel files to automated systems utilising nickel–titanium (NiTi) files. Today, the automation of root canal preparation has become standard practice in dentistry. NiTi rotary and reciprocating instruments are now indispensable tools in endodontic therapy, widely adopted by both general dentists and specialists due to their numerous advantages [1].

NiTi instruments provide superior and more predictable shaping outcomes compared to manual techniques. They allow for wider apical preparation, simplify the instrumentation process, require a shorter learning curve, and better preserve root canal anatomy and curvature. Despite these advantages, the primary drawback of NiTi instruments remains their risk of fracture [2, 3]. Fractured instrument fragments are often difficult to remove, potentially obstructing canal patency and hindering effective cleaning beyond the fracture site. This complication may compromise treatment outcomes, particularly in periapical lesions [4].

To address these challenges, advancements in NiTi alloy processing and cross‐sectional designs have significantly improved the durability and performance of endodontic instruments [5, 6]. Additionally, reciprocating motion has been shown to enhance fatigue resistance and extend the lifespan of NiTi instruments compared to continuous rotation systems [7, 8].

Despite these improvements, manufacturers recommend single use of NiTi instruments to minimise fracture risk. Repeated use causes substantial mechanical stress, leading to wear, deformation, and reduced cutting efficiency. Moreover, sterilisation cycles required for reuse may negatively impact the fracture resistance of these instruments [9, 10].

While some studies have shown that repeated use reduces shaping efficiency in simulated canals [11], others suggest that reciprocating instruments can be reused without compromising the final root canal anatomy [2, 12]. Clinical studies have also shown that reciprocating systems are safe for endodontic treatments of up to three uses in posterior teeth [2].

Currently, various thermally treated reciprocating NiTi instruments are available, with Reciproc (VDW, Munich, Germany) being the most widely used globally and the pioneer in the market. In contrast, the X1 Blue file (MK Life, Porto Alegre, Brazil) is a cost‐effective national alternative, offering similar functionality at a lower price. However, limited information is available regarding its mechanical properties and clinical performance. The X1 Blue file (X1 BF) reciprocating system includes three instruments with tip sizes of #20, #25, and #40, a 0.06 taper, a convex triangular cross‐section, and heat treatment comparable to Blue technology [13].

Given the widespread use of Reciproc and the growing interest in X1 Blue as a local alternative, comparing these files is essential for gaining critical insights into their clinical performance, including fracture risk under similar conditions.

Moreover, the reuse of NiTi instruments remains a common practice among endodontists, primarily due to the high cost of these devices. Understanding the fracture risks associated with multiple uses of these files is crucial for optimising clinical outcomes and establishing safer protocols. Therefore, this study aims to evaluate the fracture incidence of reciprocating files (Reciproc and X1 Blue) after up to three uses in anterior and posterior teeth during procedures performed by a clinical specialist.

To statistically evaluate fracture risk, we tested the null hypothesis that no difference exists in fracture incidence between Reciproc and X1 Blue files after repeated use, against the alternative hypothesis of a significant difference.

## 2 Methodology

### 2.1 Ethical Aspects

This retrospective study analysed patient records of cases treated by an endodontic specialist. Ethical approval was obtained from the Institutional Research Ethics Committee (CEP) under protocol number 68330323.5.0000.5317.

### 2.2 Study Design

The study evaluated the incidence of fractures in reciprocating files used in up to three clinical procedures. Data were extracted from the medical records of patients treated by an experienced endodontic specialist over 3 years. Findings are reported following the STROBE guidelines [14].

Patient data were anonymized before analysis by removing all identifying information. The treating dentist, also a member of the research team, blinded the medical records by eliminating names and personal details to ensure confidentiality. Access to the records was restricted; adhering to strict professional‐patient confidentiality protocols.

### 2.3 Patient Selection

The study included 1620 teeth (1346 molars, 228 premolars, and 46 anterior teeth), totalling 1720 root canals. Patients selected for inclusion underwent and completed endodontic treatment in the private office of an endodontic specialist between January 2020 and July 2023.

Inclusion criteria required patients to be over 18 years old, have at least one tooth treated endodontically with X1 Blue 25.06 (MK Life, Porto Alegre, RS, Brazil) or Reciproc R25 (VDW, Munich, Germany), and present with a closed apex. Patients who underwent multiple endodontic treatments were included numerous times in the sample, as the analysis focused on the dental element rather than the individual patient.

Exclusion criteria comprised patients with a history of fractured endodontic instruments, cases where the working length could not be achieved, treatments performed with file systems other than X1 or R25 or files with diameters other than 25.06. These restrictions ensure consistency in the comparison of fracture risks.

### 2.4 Data Collection

Data collection included the tooth number, the root canal treated, and the level at which the instrument fractured (apical, middle or coronal). The resolution of each case was documented, including whether the instrument was removed, bypassed or remained within the root canal.

Clinical records and radiographs of cases with fractured instruments were reviewed to ensure data accuracy. The final periapical radiographs, captured with a digital imaging sensor, confirmed the fractured instrument's presence, location, and size. The angle of root curvature was measured following the method described by Pruett et al. [15].

### 2.5 Technical Procedures

The study exclusively analysed root canals instrumented with X1 Blue 25.06 (MK Life, Porto Alegre, RS, Brazil) or Reciproc R25 (VDW, Munich, Germany). A standardised protocol was applied for all procedures.

The teeth were anaesthetised and isolated with a rubber dam. Coronary access was performed; root canals were explored, and the glide path was created with a K‐file #15 (Dentsply/Maillefer, Ballaigues, Switzerland). Working length (WL) was determined electronically at the apical foramen using the Romiapex A15 device (Romidan, Kiryat Ono, Israel).

Root canal preparation followed a by‐thirds instrumentation approach, employing pecking motions with continuous irrigation using 2.5% sodium hypochlorite (Biodinâmica Ltda, Ibiporã‐PR, Brazil). The apical third was prepared using the same technique until the R25 instrument reached WL. After three movement cycles, the instrument was removed, cleaned with sterile gauze, and reintroduced. Post‐instrumentation, the canals were treated with 17% ethylenediaminetetraacetic acid (EDTA), dried, and obturated during the same session.

After each procedure, instruments were inspected under magnification for signs of plastic deformation. Deformed instruments were discarded. If no deformations were observed, instruments were cleaned, sterilised in an autoclave, and reused for up to three clinical cases.

To standardise evaluations, the clinical operator underwent formal calibration for deformation detection, with all post‐procedure instrument inspections independently validated under magnification by a blinded examiner to ensure objective assessment.

### 2.6 Statistical Analysis

The comparative analysis of fracture rates between Reciproc and X1 Blue files was performed using Fisher's exact test, selected due to the small expected frequencies in our contingency table (with any expected cell count < 5). All analyses were performed using STATA 14 (StataCorp, College Station, TX), with statistical significance set at *α* = 0.05. Descriptive statistics included absolute frequencies and percentages for categorical variables. Given the low fracture incidence (*n* = 6), multivariable analyses were not performed to avoid overfitting.

## 3 Results

A total of 1720 root canals were instrumented using either the R25 Reciproc file (*n* = 1317, 76.6%) or the X1 Blue 25.06 file (*n* = 403, 23.4%). Among these, six files fractured during instrumentation, comprising four fractures (0.3%) with the R25 file and two (0.5%) with the X1 Blue file.

**FIGURE 1:**
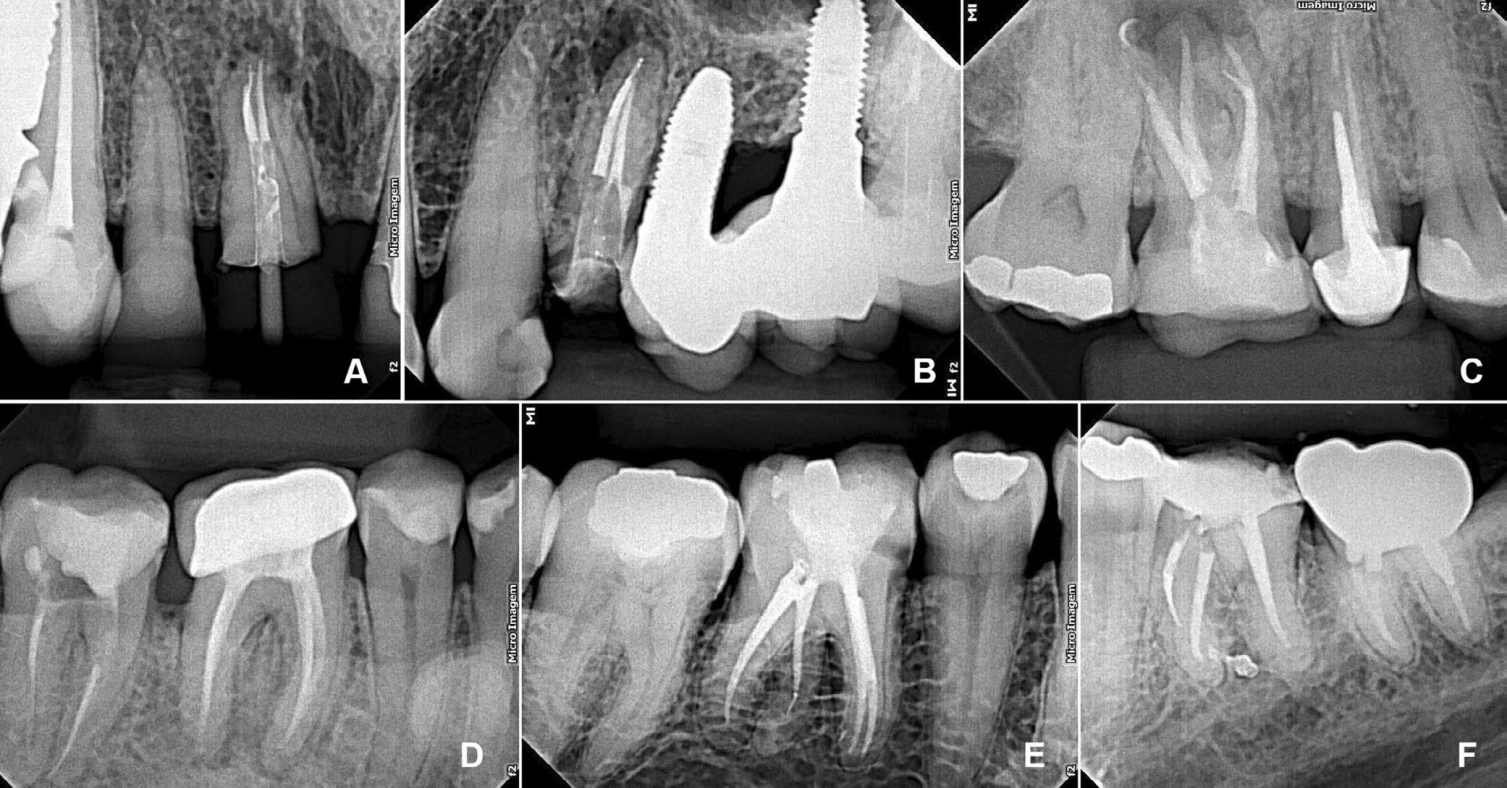
Radiographic images of the six fractures using R25 and X1 Blue instruments. (A) Fracture of an R25 instrument, used for the first time, in the apical third of the P canal in a maxillary central incisor. (B) Fractured R25 instrument in its second use, in the apical third of the P root canal of a maxillary premolar. (C) X1 Blue file, in its first use, fractured in the apical portion of the P root canal of a maxillary first molar. (D) Fractured X1 Blue in its first use in the apical third of the MB canal of a mandibular second molar. (E) Fractured R25 file, used three times, located in the apical third of the DL canal of a mandibular first molar. (F) Fractured R25 file during its first use, in the apical third of ML canal of a maxillary first molar.

Regarding the Reciproc R25 fractures, two occurred in lower molars, one in an upper central incisor, and one in an upper premolar (Figure 1). The X1 Blue file fractures were observed in one upper and one lower molar. Detailed characteristics of the fractured teeth and instruments are presented in Table 1. Fisher's exact test revealed no significant difference in fracture rates between Reciproc (0.3%) and X1 Blue (0.5%) (*p* = 0.121). A sample size of six fractured instruments resulted in a post hoc test power of 7.86% (GPower 3.1) to detect differences between the observed fracture rates (0.3% vs. 0.5%).

**TABLE 1:** Fracture frequency and detailed characteristics of root canal and instrument of each case of instrument fracture.

Group of teeth	Fracture frequency	Tooth	Root canal	Fracture level	Instrument	No. of uses	Fragment size	Case management	Angle (°)
Incisor	1	11	Palatal	Apical third	R25	1	3.63	Bypassed	13.6
Maxillary premolar	1	25	Palatal	Apical third	R25	2	2.90	Bypassed	23
Maxillar molar	1	16	Palatal	Apical third	X1	1	2.24	NRNB	47
Mandibular molar	3	36	Mesiolingual	Middle third	R25	1	3.66	NRNB	49.4
		46	Distolingual	Apical third	R25	3	1.83	NRNB	12.9
		47	Mesiobuccal	Apical third	X1	1	7.83	Removed	31.1

Abbreviation: NRNB, neither removed nor bypassed.

Of the six fractured files, one occurred in an upper central incisor, one in a premolar, and four in molars. Notably, the fracture in the upper central incisor (tooth #11) was associated with an anatomical variation, as the tooth had two root canals. All fractured fragments were in the apical third of the root canal.

The findings indicate that instrument fractures can occur even during the first clinical use, as four fractures were reported during the first use, one during the second use, and one during the third use.

Regarding fragment management, two of the six fractured instruments were successfully bypassed, enabling treatment to proceed with the fragments incorporated into the final obturation. One fragment was completely removed, while the remaining three fragments could neither be bypassed nor removed, resulting in treatment termination at the fracture site. The fragments' sizes and the root curvature's angle varied, as detailed in Table 1.

## 4 Discussion

The fracture of endodontic instruments is a significant clinical concern that demands attention from both practitioners and researchers from the Endodontic field. Understanding the factors contributing to instrument fractures and pursuing improved practices and more durable instruments is essential to minimise complications during endodontic procedures, ensuring safer and more effective patient outcomes.

The adoption of automated systems has been driven by the goal of making root canal preparation faster, simpler, and safer. However, the continuous introduction of new instrument systems into the market poses challenges for practitioners in selecting reliable options. Among Brazilian brands, MK Life (Porto Alegre, RS, Brazil) instruments stand out for their advantageous cost–benefit ratio. While Reciproc is the most globally recognised reciprocating NiTi instrument, X1 Blue (MK Life, Porto Alegre, RS, Brazil) provides an affordable alternative, delivering apparently similar performance at a reduced cost. Understanding the fracture rates of reciprocating files is crucial for clinical safety, as it directly influences the success of endodontic treatments and reduces the risk of complications during procedures. This study assessed the fracture incidence of Reciproc and X1 Blue files under reciprocating motion, comprising 1720 canals across all dental groups to ensure a diverse and representative sample. Fracture rates were observed at 0.5% for X1 Blue and 0.3% for Reciproc, reinforcing their reliability in clinical practice. While our fracture rates (0.3% Reciproc, 0.5% X1 Blue) align with literature for single‐file systems [2], the absence of a significant difference (*p* = 0.121) may reflect the files' similar metallurgy. Notably, 4/6 fractures occurred during first use, suggesting intrinsic file fatigue—not reuse—was the primary driver, contrasting with studies linking fractures to repeated sterilisation [16].

Metallurgy plays a pivotal role in the strength and performance of endodontic instruments. Differences in metal alloys and heat treatments during manufacturing influence key mechanical properties, such as flexural strength and hardness [17]. Reciproc files are produced using M‐Wire nickel–titanium, which provides higher cyclic fatigue resistance and flexibility. In contrast, X1 Blue files utilise Blue surface heat treatment and memory control, offering enhanced flexibility alongside features like an inactive tip and triangular cross‐section. Despite these metallurgical differences, this study found no apparent difference in the incidence of fractures between the two instruments, which remained low. However, the unequal number of instrumented canals between groups may have influenced these results.

Reciprocating motion has been shown to extend instrument lifespan compared to continuous rotary motion, as its alternating movements relieve stress by avoiding complete 360° rotations [7, 8]. Studies have consistently reported lower fracture rates for instruments using reciprocating motion in endodontic treatment and retreatment [2, 18, 19]. Compared with previous findings, which reported fracture rates of 0.13% to 0.43% for single‐file reciprocating systems [2, 18, 19], the present study's results remain within acceptable ranges.

While the type of motion is relevant, Gomes et al. [3] demonstrated in their meta‐analysis that kinematics plays a relatively minor role in NiTi instrument fracture, particularly for single‐use instruments. Instead, the number of uses and operator experience emerged as the strongest predictors of fracture. Their analysis highlighted a fracture incidence of 2.27% overall, differing between rotary motion (2.43%) and reciprocating motion (1.0%). These findings further emphasise the importance of operator expertise and careful instrument handling. Interestingly, most fractures in this study occurred with single‐use files, suggesting that the operator's preference to use new files in complex cases may have contributed to the overall low fracture rate. This strategy, combined with the operator's extensive clinical experience of over 10 years, likely played a role in minimising complications.

The predominance of fractures during first‐time file use (4/6 cases) challenges the conventional assumption that file fatigue from repeated sterilisation and reuse is the primary fracture risk. This finding suggests that while sterilisation cycles and repeated stress are known contributors to file fatigue [16], our data imply that manufacturing defects, operator technique or canal anatomy may play a more critical role in initial fractures. To illustrate that, two first‐use fractures found in the present study occurred in severely curved molars (Table 1), where torsional stress peaks during instrumentation [18]. This fact has a clinical implication for file selection and handling, since pre‐use inspection under magnification (as done in this study) may not detect subtle metallurgical flaws that manifest only under stress, and the single‐use protocols (already common in many clinics) could mitigate reuse‐related risks but may not prevent first‐use fractures in complex anatomies.

Most reuse studies focus on cyclic fatigue in vitro [16], but our clinical data highlight that torsional failure (e.g., from abrupt canal binding) may dominate in real‐world settings. This aligns with our findings, where most clinical fractures occurred in the apical third, where torsional stress is highest [19, 20, 21]. Future studies should correlate fracture timing with pre‐operative canal anatomy (e.g., curvature radius) and operator force metrics.

A detailed analysis of fracture location and fragment dimensions provides valuable insights into patterns and severity. Previous studies have identified the apical third as the most susceptible region for fractures, due to its limited accessibility and anatomical complexity [17, 22, 23, 24]. Iqbal et al. [24] reported that the likelihood of file separation in the apical third is 33 times higher than in the coronal third and nearly six times higher than in the middle third. These findings are consistent with this study, where all file fractures occurred in the apical third. Recognising these patterns allows clinicians to adopt techniques that minimise stress in high‐risk areas, ensuring safer preparation and improving treatment outcomes.

The fracture of an endodontic instrument is one of the accidents that can occur during endodontic treatment, potentially interfering with the cleaning and shaping of the canal, compromising periradicular healing, and thus affecting the outcome of the endodontic treatment [25]. The prognosis for vital teeth after instrument fracture is better than for necrotic teeth [26]. The prognosis is more favourable when the fracture occurs in the final stages of instrumentation, as the presence of the instrument can interfere with the disinfection of the canal system, allowing the perpetuation of infection due to reduced microbial control and minimal debridement, thus affecting the tooth's prognosis [27].

Regarding the prognosis of teeth with apical lesions, healing is less favourable when a fractured instrument is present in the canal [25]. If the fracture occurs in the apical third, the prognosis is generally favourable due to improved microbial control and the already performed debridement [4]. However, it is important to highlight that the more apical the location of the fractured instrument, the higher the probability of root perforation, which makes the tooth more susceptible to fracture after instrument removal [28].

This study also emphasised the complexity of molar anatomy, with half of the fractures observed in these teeth. This result is consistent with other studies [17, 20]; and this outcome was anticipated given the anatomical complexities of these root canals, which often feature double curvatures that may not be apparent on radiographs. The pronounced curvatures and additional canals found frequently in molars present significant challenges, increasing instrument stress during use [29]. Given most fractures occurred in curved canals, prospective trials could stratify risk by curvature severity (e.g., ≥ 30° vs. < 30°).

Even though this study provides valuable insights, certain limitations must be acknowledged. Future studies should investigate fracture rates in larger, multi‐centre cohorts with standardised torque‐controlled motors to control operational variables, while incorporating micro‐CT analysis of deformation patterns after each use to better understand fatigue accumulation in reused files. While this study provides valuable clinical data, the substantial disparity in sample sizes between groups (Reciproc: *n* = 1317 vs. X1 Blue: *n* = 403) may have limited our ability to detect statistically significant differences in fracture rates. Group sizes were unequal due to clinical practice variability, but all cases adhered to the same inclusion criteria and protocols. Fisher's Exact Test was chosen for its reliability with imbalanced data. Future randomised trials could further validate these findings. Also, the low fracture incidence limited the statistical power to identify potential differences between file systems, suggesting that larger cohorts are needed for definitive conclusions. Thus, the non‐significant *p*‐value (*p* = 0.121) should be interpreted with caution.

One possible explanation for the observed intergroup disparity is the risk of an unintentional operator‐induced selection bias. Given the limited and inconsistent evidence supporting the equivalence of X1 Blue and Reciproc files, clinicians may have been reluctant to use X1 files in complex cases due to their novelty and insufficiently validated performance. This inference further underscores the relevance of our study and highlights the need for future research. Ultimately, reliable evidence supporting cost‐effective, nationally produced instruments could broaden access to motor‐driven techniques for a greater number of clinicians, including dental students.

We acknowledge the limitations inherent to retrospective studies, including the lack of control over certain clinical variables. However, measures were implemented to mitigate these limitations and strengthen the validity of our findings: All procedures were performed by a single experienced endodontist following a strict protocol (e.g., glide path creation, irrigation regimen, WL determination), minimising variability in technique. Also, canal curvature was measured and reported [15], and all fractures occurred in the apical third, suggesting consistent anatomical challenges.

It is important to highlight that cases with previously fractured instruments or unachievable WL were excluded, reducing heterogeneity. Besides, only teeth with closed apices and 25.06 taper instruments were included, controlling for canal diameter variability. While retrospective studies cannot control for unmeasured variables (e.g., operator fatigue), the low fracture rate (*n* = 6) suggests that such factors did not systematically bias results. Pre‐flaring was not performed in any root canal treatment, as the study focused on single‐file systems (Reciproc/X1 Blue), and this consistency helps avoid additional variability.

Another limitation to be detailed is that the private practice setting may limit extrapolation to academic or low‐resource environments; where equipment, operator experience or case complexity may differ. Future multi‐centre studies could address this.

Subsequent investigations should employ balanced, prospective designs with sample size calculations based on pre‐specified clinically meaningful differences in fracture incidence. Such studies could incorporate propensity score matching to account for potential confounding variables when comparing file systems with unequal adoption rates in clinical practice. Additionally, external variables such as operator skill, while consistent in this study, may vary across different clinical settings and influence outcomes.

## 5 Conclusion

X1 Blue represents a clinically acceptable alternative to Reciproc, with comparable fracture rates. While these descriptive findings suggest both systems may be safe for limited reuse, we acknowledge that the small absolute number of fractures (*n* = 6) and unequal group sizes limited our power for inferential analysis. Clinicians must remain vigilant regarding instrument selection, handling, and usage frequency to minimise fracture risks and ensure optimal treatment outcomes.

## Author Contributions

**Elisa Korte Fortes Gollo:** methodology, investigation, writing – original draft. **Fábio de Almeida Gomes:** conceptualization, methodology, investigation, writing – original draft. **Katerine Jahnecke Pilownic:** supervision, data curation, writing – review. **Daiana Elisabeth Böttcher:** data curation, writing – review. **Carolina Clasen Vieira:** supervision, data curation, writing – original draft, writing review. **Fernanda Geraldo Pappen:** conceptualization, methodology, formal analysis, data curation, writing – review, supervision, resources, project administration. All authors have approved the final version and consent to its submission. All authors have contributed significantly, and all authors agree with the content of the manuscript.

## Disclosure

The authors have nothing to report.

## Conflicts of Interest

The authors declare no conflicts of interest.

## Data Availability

The data that support the findings of this study are available from the corresponding author upon reasonable request.

## References

[1] M. C. Cheung , O. A. Peters , and P. Parashos , “Global Survey of Endodontic Practice and Adoption of Newer Technologies,” International Endodontic Journal 56, no. 12 (2023): 1517–1533. 37800848 10.1111/iej.13982

[2] C. S. Bueno , D. P. Oliveira , R. A. Pelegrine , C. E. Fontana , D. G. Rocha , and C. E. Bueno , “Fracture Incidence of WaveOne and Reciproc Files During Root Canal Preparation of up to 3 Posterior Teeth: A Prospective Clinical Study,” Journal of Endodontics 43, no. 5 (2017): 705–708. 28343932 10.1016/j.joen.2016.12.024

[3] M. S. Gomes , R. M. Vieira , D. E. Böttcher , G. Plotino , R. K. Celeste , and G. Rossi‐Fedele , “Clinical Fracture Incidence of Rotary and Reciprocating NiTi Files: A Systematic Review and Meta‐Regression,” Australian Endodontic Journal 47, no. 2 (2021): 372–385. 33410578 10.1111/aej.12484

[4] P. Spili , P. Parashos , and H. H. Messer , “The Impact of Instrument Fracture on Outcome of Endodontic Treatment,” Journal of Endodontics 31, no. 12 (2005): 845–850. 16306815 10.1097/01.don.0000164127.62864.7c

[5] D. Abdellatif , A. Iandolo , M. Scorziello , G. Sangiovanni , and M. Pisano , “Cyclic Fatigue of Different Ni‐Ti Endodontic Rotary File Alloys: A Comprehensive Review,” Bioengineering (Basel) 11, no. 5 (2024): 499. 38790365 10.3390/bioengineering11050499 PMC11118078

[6] J. Zupanc , N. Vahdat‐Pajouh , and E. Schäfer , “New Thermomechanically Treated NiTi Alloys – A Review,” International Endodontic Journal 51, no. 10 (2018): 1088–1103. 29574784 10.1111/iej.12924

[7] A. H. Dos Reis‐Prado , L. G. Abreu , L. C. de Arantes , et al., “Influence of Sodium Hypochlorite on Cyclic Fatigue Resistance of Nickel‐Titanium Instruments: A Systematic Review and Meta‐Analysis of In Vitro Studies,” Clinical Oral Investigations 27, no. 11 (2023): 6291–6319. 37704917 10.1007/s00784-023-05243-4

[8] F. Ferreira , C. Adeodato , I. Barbosa , L. Aboud , P. Scelza , and M. Zaccaro Scelza , “Movement Kinematics and Cyclic Fatigue of NiTi Rotary Instruments: A Systematic Review,” International Endodontic Journal 50, no. 2 (2017): 143–152. 26825427 10.1111/iej.12613

[9] V. O. Peraça , S. R. Xavier , G. F. de Almeida , L. G. P. Dos Santos , E. M. Souza , and F. G. Pappen , “Effect of Number of Uses and Sterilization on the Instrumented Area and Resistance of Reciprocating Instruments,” Restorative Dentistry and Endodontics 46, no. 2 (2021): e28. 34123764 10.5395/rde.2021.46.e28 PMC8170385

[10] N. Ríos‐Osorio , J. Caviedes‐Bucheli , J. Murcia‐Celedón , et al., “Comparison of Dynamic Cyclic Fatigue Resistance of Reciproc Blue and WaveOne Gold After Sterilization and/or Immersion in Sodium Hypochlorite,” Journal of Clinical and Experimental Dentistry 16, no. 1 (2024): e1–e10. 38314336 10.4317/jced.60870 PMC10837797

[11] V. Spicciarelli , G. Corsentino , H. F. Ounsi , M. Ferrari , and S. Grandini , “Shaping Effectiveness and Surface Topography of Reciprocating Files After Multiple Simulated Uses,” Journal of Oral Science 61, no. 1 (2019): 45–52. 30713265 10.2334/josnusd.17-0311

[12] S. G. O. Barros , C. O. de Lima , V. T. L. Vieira , A. A. Neves , T. Accorsi‐Mendonça , and E. J. N. L. da Silva , “Bending Resistance and Quantitative Transportation Assessment After Multiple Uses of a Reciprocating Instrument,” Endodontic Practice Today 12 (2018): 251–256.

[13] M. E. Klymus , M. P. Alcalde , R. R. Vivan , M. V. R. Só , B. C. de Vasconselos , and M. A. H. Duarte , “Effect of Temperature on the Cyclic Fatigue Resistance of Thermally Treated Reciprocating Instruments,” Clinical Oral Investigations 23, no. 7 (2019): 3047–3052. 30397733 10.1007/s00784-018-2718-1

[14] E. von Elm , D. G. Altman , M. Egger , et al., “The Strengthening the Reporting of Observational Studies in Epidemiology (STROBE) Statement: Guidelines for Reporting Observational Studies,” Journal of Clinical Epidemiology 61 (2008): 344–349. 18313558 10.1016/j.jclinepi.2007.11.008

[15] J. P. Pruett , D. J. Clement , and D. L. Carnes, Jr. , “Cyclic Fatigue Testing of Nickel‐Titanium Endodontic Instruments,” Journal of Endodontics 23, no. 2 (1997): 77–85. 9220735 10.1016/S0099-2399(97)80250-6

[16] E. Pedullà , A. Benites , G. M. La Rosa , et al., “Cyclic Fatigue Resistance of Heat‐Treated Nickel‐Titanium Instruments After Immersion in Sodium Hypochlorite and/or Sterilization,” Journal of Endodontics 44, no. 4 (2018): 648–653. 29397218 10.1016/j.joen.2017.12.011

[17] R. S. Cunha , A. Junaid , P. Ensinas , W. Nudera , and C. E. Bueno , “Assessment of the Separation Incidence of Reciprocating WaveOne Files: A Prospective Clinical Study,” Journal of Endodontics 40, no. 7 (2014): 922–924. 24935536 10.1016/j.joen.2014.03.016

[18] S. K. Subramanian , V. Joshi , S. Kalra , and S. Adhikari , “Unveiling the Fatigue Life of NiTi Endodontic Files: An Integrated Computational‐Experimental Study,” Journal of the Mechanical Behavior of Biomedical Materials 157 (2024): 106657. 39024733 10.1016/j.jmbbm.2024.106657

[19] Y. Shen , A. M. Riyahi , L. Campbell , et al., “Effect of a Combination of Torsional and Cyclic Fatigue Preloading on the Fracture Behavior of K3 and K3XF Instruments,” Journal of Endodontics 41, no. 4 (2015): 526–530. 25459570 10.1016/j.joen.2014.10.008

[20] Y. Algarni , “Fracture Incidence of New Reciprocating Nickel‐Titanium (NiTi) Files: A Cross‐Sectional Retrospective Study,” Cureus 16, no. 8 (2024): e67762. 39323712 10.7759/cureus.67762 PMC11422514

[21] Y. Shen , H. M. Zhou , Y. F. Zheng , B. Peng , and M. Haapasalo , “Current Challenges and Concepts of the Thermomechanical Treatment of Nickel‐Titanium Instruments,” Journal of Endodontics 39, no. 2 (2013): 163–172. 23321225 10.1016/j.joen.2012.11.005

[22] H. Caballero‐Flores , C. K. Nabeshima , E. Binotto , and M. E. L. Machado , “Fracture Incidence of Instruments From a Single‐File Reciprocating System by Students in an Endodontic Graduate Programme: A Cross‐Sectional Retrospective Study,” International Endodontic Journal 52, no. 1 (2019): 13–18. 29985528 10.1111/iej.12982

[23] G. De‐Deus , G. A. Palhares , E. J. N. L. D. Silva , et al., “Effects of a Pre‐Enlargement on Fracture Incidence of Reused Reciprocating Instruments: A Clinical Study,” Brazilian Dental Journal 35 (2024): e246147. 39699503 10.1590/0103-644020246147 PMC11653994

[24] M. K. Iqbal , M. R. Kohli , and J. S. Kim , “A Retrospective Clinical Study of Incidence of Root Canal Instrument Separation in an Endodontics Graduate Program: A PennEndo Database Study,” Journal of Endodontics 32, no. 11 (2006): 1048–1052. 17055904 10.1016/j.joen.2006.03.001

[25] M. Khabiri , M. Ebrahimi , and M. R. Saei , “The Effect of Autoclave Sterilization on Resistance to Cyclic Fatigue of Hero Endodontic File #642 (6%) at Two Artificial Curvature,” Journal of Dentistry (Shiraz, Iran) 18, no. 4 (2017): 277–281. 29201971 PMC5702432

[26] Y. Shen , M. Haapasalo , G. S. Cheung , and B. Peng , “Defects in Nickel‐Titanium Instruments After Clinical Use. Part 1: Relationship Between Observed Imperfections and Factors Leading to Such Defects in a Cohort Study,” Journal of Endodontics 35, no. 1 (2009): 129–132. 19084142 10.1016/j.joen.2008.10.014

[27] M. Wefelmeier , M. Eveslage , S. Bürklein , K. Ott , and M. Kaup , “Removing Fractured Endodontic Instruments With a Modified Tube Technique Using a Light‐Curing Composite,” Journal of Endodontics 41, no. 5 (2015): 733–736. 25747379 10.1016/j.joen.2015.01.018

[28] P. Parashos and H. H. Messer , “Rotary NiTi Instrument Fracture and Its Consequences,” Journal of Endodontics 32, no. 11 (2006): 1031–1043. 17055902 10.1016/j.joen.2006.06.008

[29] J. W. Park , J. K. Lee , B. H. Ha , J. H. Choi , and H. Perinpanayagam , “Three‐Dimensional Analysis of Maxillary First Molar Mesiobuccal Root Canal Configuration and Curvature Using Micro‐Computed Tomography,” Oral Surgery, Oral Medicine, Oral Pathology, Oral Radiology, and Endodontics 108, no. 3 (2009): 437–442. 19386518 10.1016/j.tripleo.2009.01.022

